# Incidental Carcinoid Tumor of the Appendix After Appendectomy During Pregnancy

**DOI:** 10.7759/cureus.48561

**Published:** 2023-11-09

**Authors:** Jose A Smester Lopez, Jose A Garcia, Edilin Lopez, Jose A Gonzalez, Marcos Calzado Capobianco

**Affiliations:** 1 Anatomy, O&M Medical School, Santo Domingo, DOM; 2 General Surgery, Centro de Medicina Avanzada Dr. Abel Gonzalez, Santo Domingo, DOM; 3 Gynecology, Centro de Medicina Avanzada Dr. Abel Gonzalez, Santo Domingo, DOM; 4 Surgery, Baptist Health South Florida, Miami, USA; 5 Surgery, Centro de Medicina Avanzada Dr. Abel Gonzalez, Santo Domingo, DOM; 6 Research, National Institute of Diabetes (INDEN), Santo Domingo, DOM

**Keywords:** neuroendocrine neoplasm, laparotomy, carcinoid tumor, pregnancy, appendectomy

## Abstract

Neuroendocrine tumors comprise a range of neoplasms with varying spectra of origin, biological activity, clinical features, and histological appearance. In this case report, we present a pregnant 33-year-old female who was brought to the emergency department (ED) complaining of acute right iliac fossa pain accompanied by diarrhea and vomiting. Initial management showed no improvement. Lab results, clinical history, and physical exam were suggestive of appendicitis, so an exploratory minimally invasive laparoscopic exam was performed. The histopathological analysis of the excised appendix confirmed the diagnosis of acute appendicitis and periappendicitis. Incidentally, a 0.6 cm neuroendocrine tumor (carcinoid tumor) was identified on the wall of the appendiceal tip. The tumor extended at multiple points into the subserosal fat, and the serous surface and the resection margin were negative for the tumor. After seven days of the initial procedure, the patient presented with abdominal pain and a fever. An abdominal ultrasound was performed, revealing the presence of free fluid. A second exploratory laparoscopy revealed adhesions between the fallopian tubes and cecum, as well as a collection of purulent fluid. The management consisted of adhesiolysis, cavity lavage, and drainage, along with antibiotic therapy, pain management, and close monitoring of the mother's and fetus's status. The patient had a successful recovery and was discharged home a week after surgery. She gave birth to a full-term, healthy baby and remains free of tumor relapse. This case highlights the importance of obtaining histopathological interpretation of any extracted tissue during surgery. Guidelines regarding the management of carcinoids during pregnancy are not available, and when considering surgical intervention, an open or laparoscopic approach must be carefully evaluated.

## Introduction

Neuroendocrine tumors comprise a range of neoplasms with varying spectra of origin, biological activity, clinical features, and histological appearance. These neoplasms commonly arise from the digestive and pancreatic systems, causing recurrent abdominal pain [1]. However, many are discovered incidentally during endoscopy or imaging. The incidence of cancer in pregnancy is rare, complicating between 0.02% and 0.1% of pregnancies. Cancers associated with pregnancy consist of cervical, breast, and ovarian, whereas neuroendocrine tumors are rarely reported [2]. Neuroendocrine tumors can arise from most organs, but neoplasms of the appendix are scarce [3,4].

The rate of neuroendocrine neoplasms (NEN) in the United States is 6.98 per 100,000 people. In Australia, the documented rate in 2006 was 3.3 per 100,000. Norwegian registries reported a rate of 21.3 per 100,000 in 2010, while Taiwanese registries recorded a rate of 1.51 per 100,000 in 2008 [1]. Several factors may contribute to the increasing trend in NEN epidemiology; these include the greater utilization of endoscopy and advancements in the sensitivity of commonly used imaging techniques, leading to improved detection of early-stage and asymptomatic NEN cases [1]. Unlike well-differentiated neuroendocrine tumors during pregnancy, in non-pregnant individuals, neuroendocrine tumors are the most common neoplasms of the appendix. The reported incidence of appendiceal neuroendocrine tumors is 3 to 9 per 1000 appendectomies [5-7]. Appendiceal carcinoids, a type of tumor, accounted for 18.9% of all carcinoid tumors and showed a significant prevalence among females (male-to-female ratio: 0.47). The incidence rates, adjusted for age, were 1.7 times higher in women compared to men. On average, appendiceal carcinoids were diagnosed at an earlier age (average age: 42.2 years) compared to other gastrointestinal carcinoids (62.9 years) or non-carcinoid tumors of the appendix (61.9 years) [5].

The earliest malignant tumor of the appendix was described in 1882, and the first reports about appendiceal tumors and pregnancy are dated from 1965 [8]. The etiology of de novo neuroendocrine tumors is not fully comprehensible, but it is possibly affiliated with spontaneous regression, including an immunological response, activation of proapoptotic pathways, cytokines and growth factors, and psychological mechanisms [2]. Most diagnoses are made during adulthood, with a median age at diagnosis of 29.8 years [8]. We reported a case of a biologically inactive, well-differentiated, low-grade neuroendocrine tumor of appendiceal origin that was found incidentally during an exploratory laparoscopy.

## Case presentation

A 33-year-old female at 19 weeks of gestation, with a prior surgical history of cholecystectomy 11 weeks before, was brought to the emergency department (ED) complaining of acute right iliac fossa pain accompanied by diarrhea and vomiting. An urgent pelvic ultrasound revealed a gravid uterus adequate for gestational age, with no pathological findings in the abdomen or pelvis. Initial management with IV fluids, ceftriaxone 1 gram twice a day, and clindamycin 300 mg every 8 hours for three days showed no improvement. Magnetic resonance imaging (MRI) was not available at the time, but lab results, clinical history, and a physical exam were suggestive of appendicitis, so an exploratory minimally invasive laparoscopic exam was performed. During the procedure, abundant purulent fluid was found in the pelvic region and right paracolic side, as well as an inflamed, distended appendix with abundant fibrin but without perforation. Therefore, an appendectomy and cavity drainage were performed.

**Figure 1:**
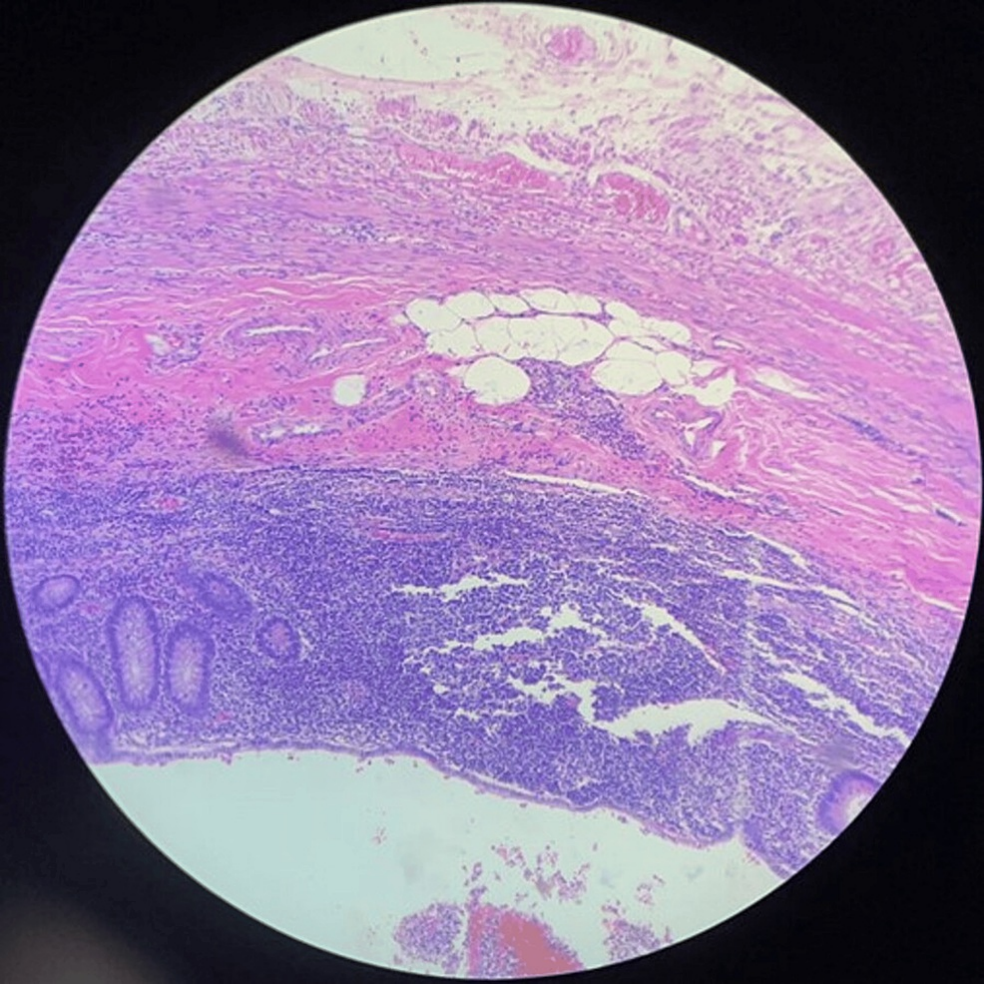
Histopathological sections of the tip of the appendix on a Pietri slide The presence of inflammatory cells is identified, in addition to tumor cells.

The histopathological analysis of the excised appendix, as shown in Figure 1, confirmed the diagnosis of acute appendicitis and periappendicitis. The specimen is received in formalin solution and consists of a vermiform appendix measuring 6.0 cm in length and 1.2 cm in mean maximum diameter in the distal third. The serosal surface is grayish and covered in areas by a thick, yellowish exudate. It includes the corresponding mesoappendix with a maximum width of 2.2 cm. Black ink is applied to the resection margin, and blue ink is applied to the serosal surface of the appendix.

**Figure 2:**
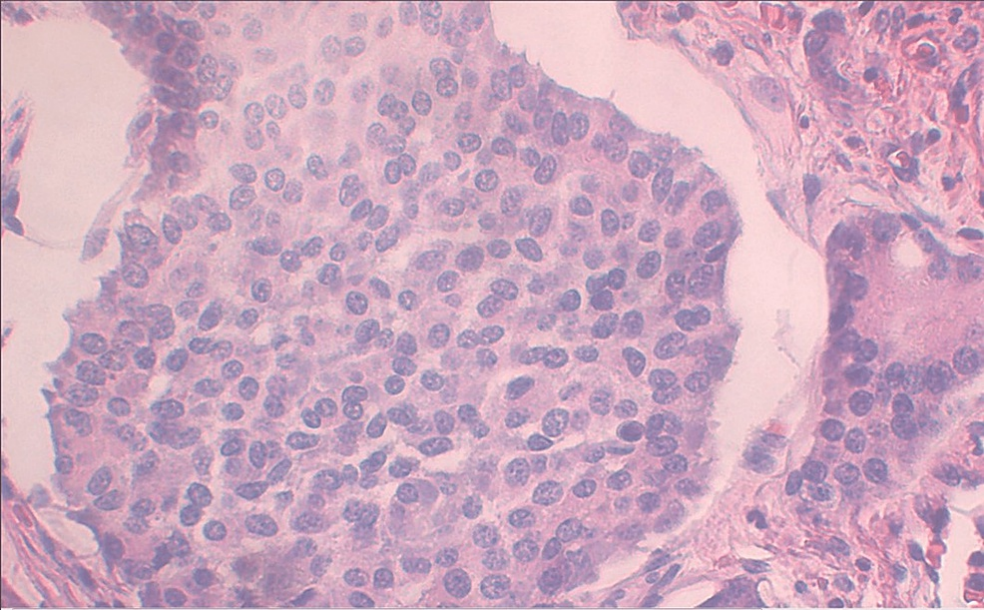
Appendiceal carcinoid tumor The tumor cell specimens are comprised of uniform, round, and polygonal cells with eosinophilic cytoplasm, arranged in nests and trabecular patterns. These cells possess centrally located nuclei and stippled chromatin (a pattern referred to as "salt and pepper"), which represents a characteristic manifestation of carcinoid tumor cells.

Incidentally, a neuroendocrine tumor (carcinoid tumor) was identified on the wall of the appendiceal tip. Maximum diameter of 0.6 cm (Figure 2). Tumor necrosis and mitotic activity were identified. The tumor extended at multiple points into the subserosal fat, and the serous surface and the resection margin were negative for the tumor.

The patient was discharged on post-operative day two without complications, and no drain was placed. After seven days of the initial procedure, the patient showed presenting abdominal pain and fever. An abdominal ultrasound was performed, which revealed the presence of free fluid. However, percutaneous drainage was not possible due to the position of the bowels. A second exploratory laparoscopy revealed adhesions between the fallopian tubes and cecum and a collection of purulent fluid. The management consisted of adhesiolysis, cavity lavage, and drainage. In addition, the patient received antibiotic therapy, including clindamycin 450 mg three times a day for seven days and cefuroxime 250 mg every 12 hours for 10 days. Pain management measures were implemented, and close monitoring of the mother and fetus's status was maintained. The patient had a successful recovery and was discharged home one week after surgery.

Weekly follow-up was conducted for a period of four weeks, during which no further complications related to her pregnancy were observed. She gave birth to a full-term healthy baby. Additional follow-up visits were scheduled for the first and second years after surgery, and during these visits, no symptoms or signs were reported. Furthermore, there were no alterations observed in subsequent tumoral and inflammatory markers.

## Discussion

Appendiceal neuroendocrine tumors (NETs) can be categorized into two main types: typical carcinoids and goblet cell carcinoids. These types of tumors make up only approximately 5% of neuroendocrine tumors found in the gastrointestinal tract. According to the pathology description in this case, a rare, typical carcinoid was diagnosed in this patient [3]. However, it is worth noting that all the reported cases of neuroendocrine tumors during pregnancy have been non-secretory neuroendocrine tumors, like the present case [3].

Laparoscopic appendectomy is a common procedure in the surgical realm that can be performed during all trimesters of pregnancy. The advantages of this approach help avoid high complication rates and result in shorter hospital stays compared to an open appendectomy [9]. However, the optimal surgical intervention for appendiceal neuroendocrine tumors (NETs), also known as carcinoid tumors, remains controversial. Since these tumors are typically found incidentally in postoperative appendectomy specimens, a decision must be made whether to perform a right colectomy or not. A colectomy involves the removal of the colon and the lymph nodes along the ileocolic and right colic arteries, as well as any residual tissue that may be present at the base of the appendix or in the mesoappendix, to minimize the risk of metastasis [2,3].

Well-differentiated neuroendocrine tumors are relatively rare, but their incidence has increased over time in the United States and globally [10]. The annual incidence rate is slightly higher for African Americans compared to white Americans (6.46 versus 4.6 per 100,000), and the incidence is slightly higher in males than females (4.97 versus 4.49 per 100,000). Within the gastrointestinal tract, most neuroendocrine tumors arise in the small intestine (45%, most commonly in the ileum), followed by the rectum, appendix, colon, and stomach [11]. It is rare to find appendiceal carcinoid tumors during pregnancy.

There are no reported studies that establish an association between acute appendicitis and incidental carcinoid tumors. Carcinoid tumors in the appendix are typically submucosal and tend to obstruct the distal third of the appendix. They are often asymptomatic, which makes early diagnosis challenging [12]. Appendiceal carcinoid tumors are generally diagnosed more frequently in females than in males. There are no classic symptoms specifically attributed to this tumor or that it presents as acute appendicitis, such as nausea, acute abdominal pain, or emesis [13,14].

Appendiceal carcinoids have a slow growth rate, and the overall prognosis is excellent [15]. Tumor size is a reliable indicator for assessing the malignant potential of these lesions. In most cases, appendiceal carcinoids have a diameter of less than 1 cm, and treatment with appendectomy alone is usually sufficient as these tumors rarely metastasize. However, tumors measuring 2 cm or larger in diameter may have a higher potential for metastasis, and patients with tumors of this size may require a right hemicolectomy [14]. All the characteristics of the patient's tumor, including its small size (0.6 cm), non-secretory nature, and location at the tip of the appendix, indicated a favorable prognosis. These factors justified a less aggressive surgical approach, as the margins were clear, and the decision was made to avoid colectomy while ensuring continuous monitoring and supervision.

The prognosis and treatment of appendiceal carcinoid tumors are influenced by various factors, including tumor size and location [12,13]. According to the National Comprehensive Cancer Network 2013 Guidelines, postoperative follow-ups are recommended for patients who have undergone excision of appendiceal carcinoids, particularly for tumors larger than 2 cm in diameter. The follow-up protocol typically involves regular clinical history and physical examinations every 3 to 12 months in the first few years post-resection and then every 6 to 12 months thereafter for up to 10 years. Additional monitoring may include imaging studies or laboratory markers such as 5-hydroxyindoleacetic acid or chromogranin A [12,13]. Regrettably, the specific markers needed to confirm the pathological diagnosis of the tumor were unavailable. However, based on the information we have, the initial follow-up markers from the oncology department yielded negative results. It is crucial to emphasize that the long-term prognosis for appendiceal carcinoid tumors is generally excellent, and the primary treatment typically involves appendectomy.

A large cohort study reported that patients with local disease (without metastasis) have a five-year survival rate of 92%. In comparison, those with regional metastases (spread to nearby lymph nodes or tissues) have a five-year survival rate of 81%. The minority of patients with distant metastases (spread to distant organs) have a significantly lower five-year survival rate of 31% [14]. These findings highlight the importance of early detection and treatment to improve the prognosis for patients with appendiceal carcinoid tumors.

Our case highlights the importance of closely monitoring histopathological findings after an emergent appendectomy. Follow-up for neuroendocrine tumors typically involves regular serum marker tests and imaging. These assessments are usually performed three months after the surgery, followed by subsequent evaluations every six months for 1 to 3 years. If necessary, annual blood tests and imaging studies can be conducted thereafter [8]. Our patient's case is unique due to the low incidence of tumors arising during pregnancy (0.02% to 0.1%). Being a 19-week-pregnant female, she was diagnosed with an extremely rare appendiceal neuroendocrine tumor. Prompt and effective treatment was provided through an immediate appendectomy and exploratory laparotomy.

## Conclusions

Appendiceal neuroendocrine neoplasms are uncommon and typically exhibit less aggressive behavior compared to other gastrointestinal tumors. In this case, we present the incidental discovery of a biologically inactive neuroendocrine tumor during routine histopathological examination following an appendectomy in a young female at 19 weeks of gestation. The literature reports that abdominal pain and the presence of an abdominal mass are the most frequent presentations for diagnosing this type of tumor. However, inflammatory signs observed during surgical evaluation and pathological analysis assured us that appendicitis was the primary cause of this patient's presentation, and the concomitant low-sized carcinoid tumor of the appendix was merely present incidentally in this pregnant woman. We believe that pregnancy is not related to the appearance or growth of this tumor; therefore, we were able to diagnose it incidentally in the patient. This highlights the significance of a thorough histopathological analysis in identifying unexpected diseases and facilitating prompt investigation and management. A multidisciplinary approach is crucial in cases like this to ensure the patient's good prognosis.
